# Serum Interleukin-6 as an Inflammatory Biomarker Associated with HBV Viral Load in HBsAg-Positive Chronic Hepatitis B

**DOI:** 10.3390/diseases14060209

**Published:** 2026-06-10

**Authors:** Jayakrishna Pamarthi, Sugan Panneerselvam, Nanda Amarnath Rajesh, Venkataratna Bharat Gangireddy, Mohanram Murugan, Leela Kakithara Vajaravelu, Jayaprakash Thulukanam, Mansour Alanazi, Janardanan Subramonia Kumar

**Affiliations:** 1 Department of Microbiology, SRM Medical College Hospital and Research Centre, Faculty of Medicine and Health Sciences, SRM Institute of Science and Technology, Kattankulathur, Chengalpattu 603203, Tamil Nadu, India; pamarthijai@gmail.com (J.P.); suganfr@gmail.com (S.P.);; 2 Medical Gastroenterology, SRM Medical College Hospital and Research Centre, Faculty of Medicine and Health Sciences, SRM Institute of Science and Technology, Kattankulathur, Chengalpattu 603203, Tamil Nadu, India; rajesha@srmist.edu.in; 3 Department of General Medicine, SRM Medical College Hospital and Research Centre, Faculty of Medicine and Health Sciences, SRM Institute of Science and Technology, Kattankulathur, Chengalpattu 603203, Tamil Nadu, Indiamm0244@srmist.edu.in (M.M.); 4 Department of Internal Medicine, College of Medicine, Northern Border University, Arar 91431, Saudi Arabia

**Keywords:** hepatitis B virus, interleukin-6, real-time polymerase chain reaction, hepatitis E antigen

## Abstract

Background: Chronic hepatitis B virus (HBV) infection remains a major global health challenge and a leading cause of liver cirrhosis and hepatocellular carcinoma. Interleukin-6 (IL-6), a key pro-inflammatory cytokine, plays an important role in immune regulation and hepatic inflammation. However, its relationship with HBV viral load and disease severity remains incompletely understood. Methods: A hospital-based cross-sectional study was conducted among 293 HBsAg-positive patients. Serum IL-6 levels were measured using ELISA, and HBV DNA was quantified using real-time quantitative PCR. Patients were stratified according to viral load. Statistical analyses included non-parametric tests, Spearman correlation, principal component analysis (PCA), and multiple linear regression. Results: The median age was 45 years (IQR: 34–57), among which 54.6% were male. The median HBV DNA was 3.37 log_10_ IU/mL (IQR: 2.45–3.75), and IL-6 concentration was 2.38 log_10_ pg/mL (IQR: 2.21–2.49). IL-6 levels increased significantly across viral load categories (*p* < 0.001) and were higher in HBeAg-positive patients (*p* = 0.002), with no significant differences across age, sex, or cirrhosis. IL-6 levels correlated with HBV DNA (r = 0.40, *p* < 0.001). PCA identified distinct viral-inflammatory and biochemical axes. Regression analysis confirmed HBV DNA as the significant independent predictor (β = 0.461, *p* < 0.001; adjusted R^2^ = 0.206). Conclusion: IL-6 was closely associated with HBV DNA levels, while the association with conventional biochemical markers of hepatocellular injury was significantly less in this cohort, suggesting that IL-6 may serve as an adjunct biomarker of disease activity in patients with chronic hepatitis B.

## 1. Introduction

Hepatitis B virus (HBV) infection remains a major global health concern, affecting approximately 254 million people worldwide, with an estimated hepatitis B surface antigen (HBsAg) seroprevalence of 3.2%. It contributes substantially to the global burden of chronic liver diseases, including cirrhosis and hepatocellular carcinoma (HCC) [1]. The host immunological response, specifically the balance between pro-inflammatory and anti-inflammatory cytokines, plays a crucial role in controlling the clinical course of HBV infection [2,3].

Among the cytokines involved in HBV immunopathogenesis, interleukin-6 (IL-6) is a well-recognized pro-inflammatory mediator that plays an important role in regulating immune responses in patients with chronic hepatitis B (CHB) and HCC [4]. Experimental investigations in vitro and in animal models further confirm the function of IL-6 in the onset and progression of liver cancer, most likely because of its participation in inducing hepatic damage and compensatory growth [5]. IL-6 is produced by a variety of cells, including hepatocytes, Kupffer cells, and immune cells, in response to viral infections and inflammatory stimuli [6]. Functionally, it regulates T-cell differentiation, B-cell maturity, and acute-phase activity and thus contributes to antiviral defense and disease progression [7,8]. Elevated IL-6 levels have been associated with liver inflammation, fibrosis, and progression to HCC in the context of chronic HBV infection [9]. Therefore, increased IL-6 levels may represent ongoing hepatic necroinflammation and might be associated with disease severity in HBV-infected people.

HBV viral load is a critical determinant of disease activity and progression. High HBV DNA levels are typically associated with increased immune activation and hepatocellular injury, while low viral load may indicate effective immune control or an immune-tolerant phase of infection [10]. However, the relationship between IL-6 expression and HBV viral load remains complex and incompletely understood. Some studies have reported that IL-6 levels are increased in patients with high HBV DNA, possibly reflecting enhanced immune activation and liver damage [11]. Conversely, other studies suggest that IL-6 may suppress HBV replication through STAT3-mediated mechanisms [12]. The clinical course of HBV infection is largely determined by the interaction between viral replication and host immune responses, particularly cytokine-mediated inflammation [13].

IL-6 is a key pro-inflammatory cytokine involved in immune regulation, hepatocyte injury, and fibrogenesis. Elevated IL-6 levels have been reported in chronic hepatitis B (CHB) and HCC, suggesting its role in disease progression. However, the relationship between IL-6 levels and HBV viral load remains unclear and potentially bidirectional, with evidence suggesting both pro-inflammatory and antiviral roles. Despite increasing interest in IL-6 as a biomarker, its association with viral replication dynamics and disease severity across different viral load strata has not been fully elucidated. Therefore, this study aimed to evaluate IL-6 levels in HBV-infected patients stratified by DNA viral load and to assess their association with disease severity and biochemical parameters.

## 2. Materials and Methods

### 2.1. Study Design and Participants

The present study was a hospital-based cross-sectional study conducted between 1 January 2024 and 31 December 2025 at the Department of Medical Gastroenterology, SRM Medical College Hospital, and SRM Global Hospital, Kattankulathur, Tamil Nadu, India. A total of 293 patients were included, all of whom were HBsAg-positive for ≥6 months and aged ≥18 years. Patients with autoimmune diseases or co-infections, including HIV, HCV, and tuberculosis, were excluded from the study. The study was approved by the Institutional Ethics Committee (EC No. 8854) and adhered to the ethical principles of the Declaration of Helsinki [14].

### 2.2. IL-6, HBsAg, HBeAg, and HBV DNA Detection

Serum IL-6 levels were quantified using ELISA (Cat# KB1068, Krishgen Biosystems; Cerritos, CA, USA) and adhered to the manufacturer’s instructions. Briefly, 100 μL of standards and serum samples were added to microplate wells and incubated for 2 h at room temperature, followed by sequential incubation with detection antibody (1 h) and streptavidin–HRP conjugate (15 min), with washing steps between each stage. After the addition of TMB substrate and incubation in the dark for 30 min, the reaction was stopped, and absorbance was measured at 450 nm. Serum HBsAg and HBeAg levels were quantified using chemiluminescent microparticle immunoassays (CMIA) on the Abbott Architect i1000SR platform according to the manufacturer’s instructions(Abbott, Irving, TX, USA; ) (Cat#105067, Cat#105228,Roche; Basel, Switzerland). HBV DNA quantification was performed using the GeneXpert HBV Viral Load (Cat#GXHBV-VL-CE-100, Cepheid, Sunnyvale, CA, USA, ) assay. Briefly, 1 mL of plasma or serum was directly loaded into a self-contained cartridge containing all reagents required for automated real-time PCR amplification and detection. The assay has a quantification range of 10 to 1 × 10^9^ IU/mL (equivalent to 1.0 to 9.0 Log IU/mL) and a limit of detection (LOD) of 3.20 IU/mL for plasma and 5.99 IU/mL for serum as per the manufacturer’s instructions [15].

### 2.3. Statistical Analysis

Statistical analyses were performed using IBM SPSS Statistics version 25 and GraphPad Prism 8.0. Continuous variables are expressed as mean ± standard deviation or median with interquartile range after assessing normality using the Shapiro–Wilk test. Skewed variables, including IL-6 and HBV DNA, were log10-transformed. Categorical variables were summarized as frequencies and percentages, and group comparisons were performed using the chi-square or Fisher’s exact test, while non-parametric tests (Mann–Whitney U) were applied for continuous variables. Associations between variables were evaluated using Spearman’s correlation analysis. Principal component analysis (PCA) with varimax rotation was conducted to identify clustering patterns, with sampling adequacy assessed using the Kaiser–Meyer–Olkin test and Bartlett’s test of sphericity. Multiple linear regression analysis was performed to determine independent predictors of IL-6 levels, with model fit assessed using R^2^ and regression coefficients reported with 95% confidence intervals. A two-tailed *p*-value < 0.05 was considered statistically significant.

## 3. Results

A total of 293 patients with chronic hepatitis B were included in the analysis, including 160 males (54.6%) and 133 females (45.4%). The median age was 45 years (IQR: 34–57), with the largest proportion of individuals falling within the 41–50 years age group (24.9%), followed by 31–40 years (23.2%) and those aged above 61 years (19.4%). Patients aged below 20 years constituted only 1.3% of the cohort. Based on HBV DNA levels, 134 patients (45.7%) had viral loads <2000 IU/mL, 138 (47.1%) had levels between 2001 and 200,000 IU/mL, and 21 patients (7.2%) had viral loads >200,000 IU/mL. HBeAg positivity was observed in 9 patients (3%), while the majority were HBeAg negative (97%). The median total bilirubin levels were 0.74 mg/dL (IQR: 0.52–1.20), and direct bilirubin levels were 0.15 mg/dL (IQR: 0.10–0.30). Median liver enzyme values were 27 IU/L (IQR: 20–48) for AST and 24 IU/L (IQR: 16–42) for ALT. Cholestatic markers showed median ALP and GGT levels of 72 IU/L (IQR: 59–94) and 23 IU/L (IQR: 15–37), respectively. Median total protein, albumin, and globulin levels were 6.9 g/dL (IQR: 6.3–9.4), 3.8 g/dL (IQR: 3.3–4.2), and 3.0 g/dL (IQR: 2.8–3.4), respectively. Renal and electrolyte parameters were largely within normal ranges, with a median blood urea of 21 mg/dL (IQR: 16.5–27), creatinine of 0.7 mg/dL (IQR: 0.6–0.9), sodium of 135 mmol/L (IQR: 133–137), and chloride of 102 mmol/L (IQR: 99–105). Coagulation profiles showed a median prothrombin time of 15 s (IQR: 14.1–16.1), INR of 1.1 (IQR: 1.0–1.2), and APTT of 28.4 s (IQR: 26.5–31.1). Notably, 3.37 log10 IU/mL (IQR: 2.45–3.75) was the median HBV viral load. The median log10-transformed IL-6 level was 2.38 pg/mL (IQR: 2.21–2.49). Platelet counts had a median value of 241,000/µL (IQR: 195,500–299,000). Fibrosis assessment revealed median FIB-4 and APRI scores of 1.09 (IQR: 0.67–1.69) and 0.34 (IQR: 0.23–0.69), respectively, while the median PAGE-B score was 6 (IQR: 1–7) (Table 1). The details regarding clinical manifestation are provided in Supplementary Table S1.

### 3.1. Clinical and Biochemical Variations Across HBV Viral Load Categories 

Stratification of the 293 patients according to HBV DNA levels (<2000, 2000–200,000, and >200,000 IU/mL) demonstrated a strong association between increasing viral load and disease severity. Higher HBV DNA levels were more frequently observed among middle-aged adults (31–60 years) and males, with a significant sex-based difference across viral load categories (*p* = 0.04). Biochemical indicators of liver injury worsened progressively with rising viral burden. Abnormal total and direct bilirubin levels, along with elevated AST and ALT, were significantly more prevalent in patients with increased HBV DNA levels (all *p* = 0.001), reflecting increasing hepatocellular injury. In contrast, cholestatic enzymes (ALP and GGT), serum proteins, creatinine, and electrolyte levels did not differ significantly among the viral load groups. Blood urea abnormalities were more common in patients with higher viral loads (*p* = 0.04). Coagulation parameters also reflected declining hepatic synthetic function, with prolonged prothrombin time and abnormal APTT significantly linked with higher HBV DNA levels (*p* = 0.001), while INR showed a non-significant upward trend. Platelet abnormalities were more frequent in high viral load groups, suggesting evolving portal hypertension. Virological and fibrosis markers were strongly linked to viral load. HBeAg negativity increased significantly with HBV DNA (*p* = 0.001), and fibrosis scores (FIB-4, PAGE-B, and APRI) showed a stepwise rise with increasing viral burden (*p* ≤ 0.01). Overall, higher HBV DNA levels were strongly linked with worsened liver damage, poorer coagulation, and increased fibrosis, highlighting viral load as a critical predictor of disease development (Table 2).

### 3.2. Association of IL-6 with Clinical, Demographic, and Virological Factors

**Figure 1:**
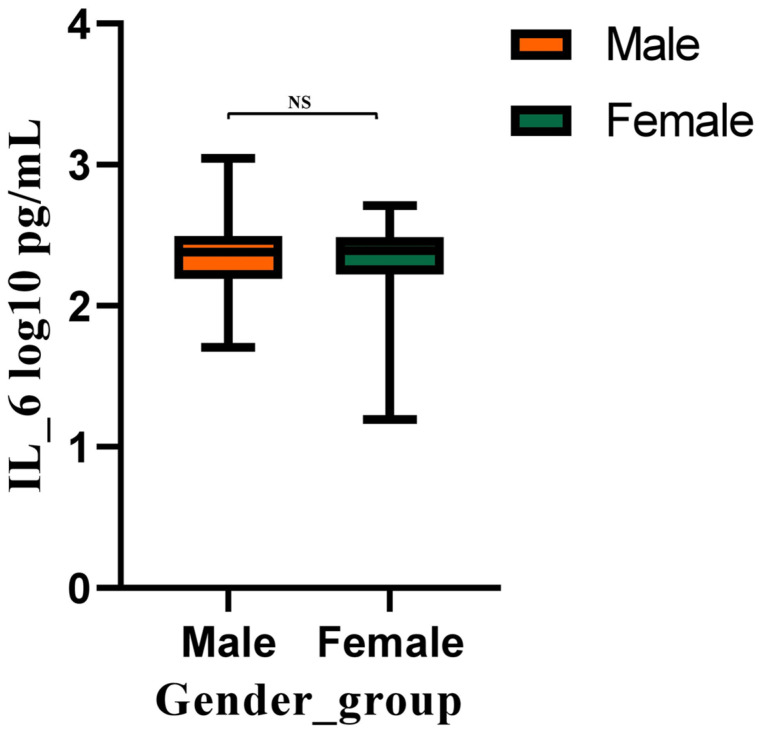
Comparison of IL-6 concentrations between male and female HBV patients. **Note:** Serum IL-6 levels (log10 pg/mL) did not differ significantly between male and female patients (NS, no significance).

**Figure 2:**
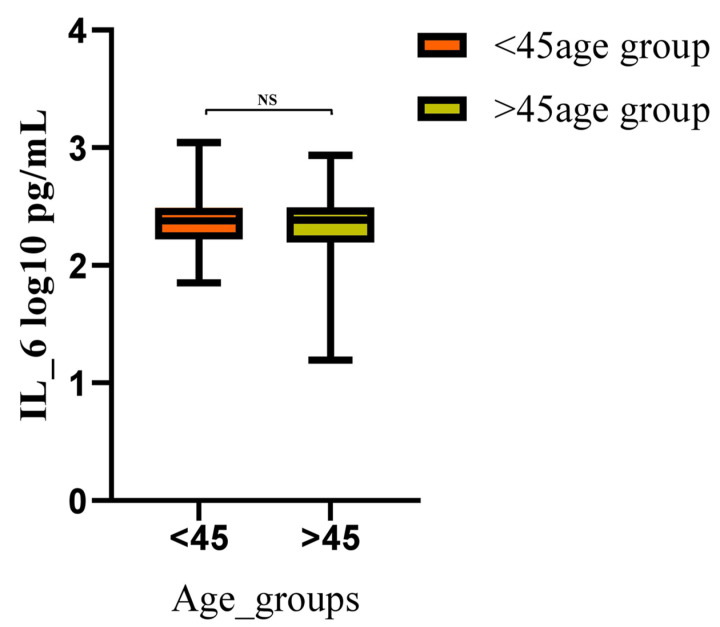
Comparison of IL-6 concentrations (pg/mL) between younger (<45 years) and older (>45 years) HBV patients. Note: Serum IL-6 levels (log10 pg/mL) showed no significant difference between patients aged <45 years and >45 years (NS, no significance).

Group comparisons were performed using the Mann–Whitney U test. No significant differences in IL-6 levels were observed across sex or age groups. Mean rank values were comparable between males and females (147.05 vs. 146.94; Z = −0.012; *p* = 0.90) and between patients aged <45 and ≥45 years (147.85 vs. 146.14; Z = −0.17; *p* = 0.86), indicating that IL-6 concentrations were independent of these demographic factors (Figure 1 and Figure 2).

**Figure 3:**
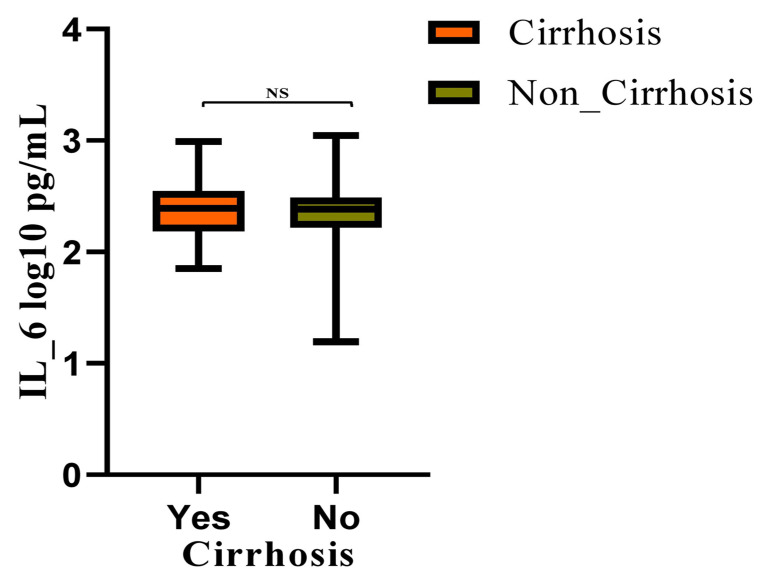
Comparison of serum IL-6 levels between cirrhotic and non-cirrhotic chronic hepatitis B patients. Note: Serum IL-6 levels (log10 pg/mL) did not differ significantly between cirrhotic and non-cirrhotic patients (NS, no significance).

Similarly, IL-6 levels were not significant between cirrhotic and non-cirrhotic patients (mean rank: 152.00 vs. 146.45; U = 3683; Z = −0.34; *p* = 0.70), suggesting no association with cirrhosis status (Figure 3).

**Figure 4:**
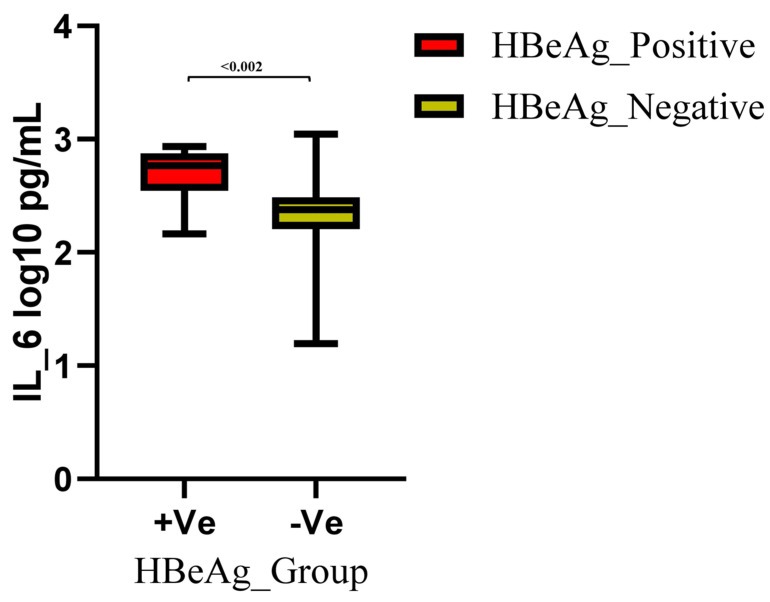
Comparison of IL-6 concentrations (pg/mL) between HBeAg-positive and HBeAg-negative HBV patients. Note. Serum IL-6 levels (log10 pg/mL) were significantly higher in HBeAg-positive patients compared to HBeAg-negative patients (*p* = 0.002).

In contrast, IL-6 levels were higher among HBeAg-positive patients than HBeAg-negative patients (mean rank: 149.76 vs. 59.78; U = 493; Z = −3.14; *p* = 0.002). However, this finding should be interpreted with caution due to the small number of HBeAg-positive participants (*n* = 9) in this study population (Figure 4).

**Figure 5:**
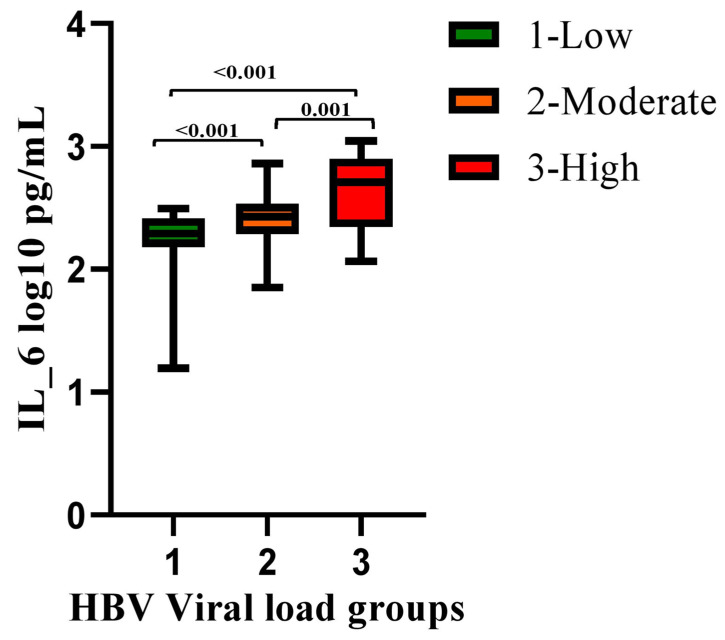
Comparison of IL-6 concentrations between HBV DNA groups. Note: Serum IL-6 levels (log10 pg/mL) showed a substantial rise across HBV viral load categories (low, moderate, and high), with elevated levels noted in moderate and high groups relative to low (*p* < 0.001) and in high compared to moderate (*p* = 0.001).

Pairwise comparisons across HBV DNA categories demonstrated a significant stepwise increase in IL-6 levels with rising viral load. IL-6 levels were higher in the moderate viral load group compared with the low viral load group (mean rank: 162.07 vs. 110.17; Z = −5.44; *p* < 0.001) and further increased in the high viral load group compared with both moderate (mean rank: 111.24 vs. 75.25; Z = −3.34; *p* = 0.001) and low viral load groups (mean rank: 119.12 vs. 71.56; Z = −4.52; *p* < 0.001) (Figure 5; Supplementary Table S3).

### 3.3. Multivariate Analysis of IL-6 Associations

**Figure 6:**
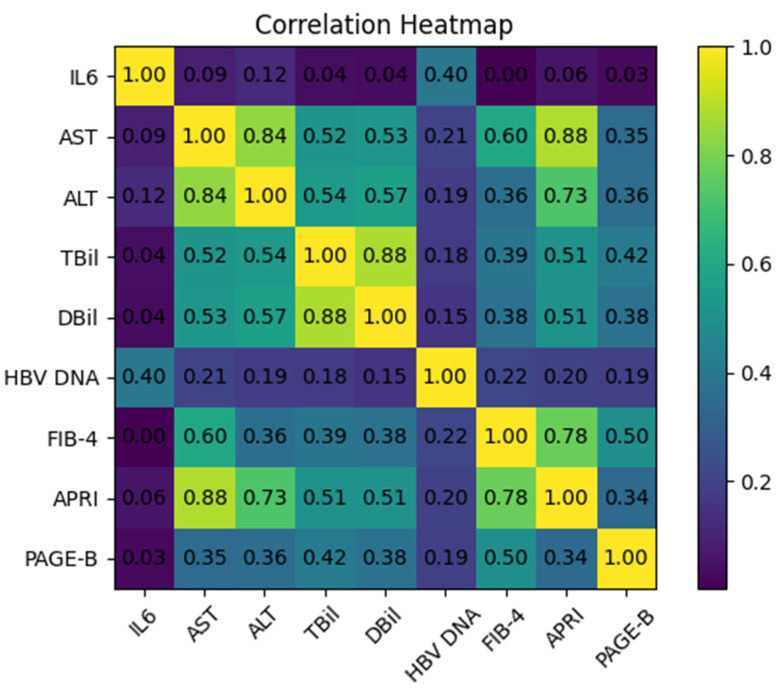
Spearman correlation matrix of IL-6, liver biochemical parameters, viral load, and fibrosis indices in patients with chronic hepatitis B. Note: Heatmap showing Spearman correlations among IL-6, HBV DNA, biochemical markers, and fibrosis indices. IL-6 correlates with HBV DNA but not with conventional biochemical markers of hepatocellular injury or fibrosis markers, while strong interrelationships are observed among biochemical and fibrosis parameters.

Serum IL-6 levels showed a moderate positive correlation with HBV DNA (r = 0.40), indicating an association with viral replication, while exhibiting weak or negligible correlations with liver enzymes (AST, ALT), bilirubin levels, and fibrosis indices (FIB-4, APRI, and PAGE-B), suggesting relative independence from conventional biochemical markers of hepatocellular injury and fibrosis. In contrast, strong correlations were observed among biochemical and fibrosis parameters, including AST with ALT (r = 0.84), total bilirubin with direct bilirubin (r = 0.88), and APRI with AST (r = 0.88) and ALT (r = 0.73). FIB-4 also correlated strongly with APRI (r = 0.78) and moderately with AST (r = 0.60), while PAGE-B demonstrated moderate associations with FIB-4 and bilirubin levels (Figure 6). PCA was performed to explore underlying relationships among virological, biochemical, and fibrosis-related variables. The Kaiser–Meyer–Olkin statistic (KMO = 0.502) and Bartlett’s test of sphericity (*p* < 0.001) supported the suitability of the dataset for PCA. Two principal components were identified. The first component (PC1) was primarily driven by conventional biochemical markers of hepatocellular injury, including AST, ALT, total bilirubin, and direct bilirubin, indicating a biochemical liver injury axis. The second component (PC2) was characterized by clustering of IL-6 and HBV DNA, suggesting that these variables share a common pattern of variation distinct from conventional liver injury markers. This separation indicates that IL-6 levels were more closely associated with HBV DNA levels in this cohort (Supplementary Figure S1). To further evaluate independent associations, multiple linear regression analysis was performed. The overall model was statistically significant (F = 13.65, *p* < 0.001), explaining 22.3% of the variance in IL-6 levels (R^2^ = 0.223; adjusted R^2^ = 0.206). The modest proportion of variance explained by the model indicates that additional clinically relevant factors, including antiviral treatment status, HBV disease phase, and coexisting inflammatory conditions, may contribute to variability in IL-6 levels. HBV DNA (log10 IU/mL) emerged as the strongest independent predictor of IL-6 levels (β = 0.461, *p* < 0.001), while ALT showed a weak but statistically significant association (β = 0.194, *p* = 0.036). In contrast, AST, bilirubin parameters, and FIB-4 were not significantly associated with IL-6 after adjustment (all *p* > 0.05) (Supplementary Table S4). However, these findings indicate that IL-6 showed stronger statistical associations with HBV DNA levels than with conventional biochemical markers of liver injury or fibrosis.

## 4. Discussion

In this study, stratification of CHB patients according to HBV DNA levels revealed notable associations among viral burden, clinical characteristics, and inflammatory responses. Higher viral loads were predominantly observed among individuals aged 31–50 years, supporting previous evidence that viral replication is more active in younger and middle-aged adults due to dynamic immune–viral interactions [16]. Increasing HBV DNA levels were significantly associated with abnormalities in key biochemical markers of hepatocellular injury, including bilirubin (total and direct), AST and ALT, indicating progressive hepatocellular damage with rising viral replication. In contrast, cholestatic enzymes, serum proteins, creatinine, and electrolytes did not differ significantly across viral load groups. Blood urea levels showed a modest but significant increase with higher viral load, suggesting early metabolic involvement. Overall, higher HBV DNA levels were consistently associated with worsening liver biochemistry, reflecting increased disease activity, in line with previous reports linking elevated viral load to hepatic dysfunction [17]. The strong association between HBeAg positivity and high viral load observed in this cohort aligns with the established natural history of HBV infection, wherein HBeAg serves as a surrogate marker of active viral transcription. Additionally, fibrosis indices (FIB-4, APRI, and PAGE-B) increased progressively across HBV DNA categories, indicating that higher HBV DNA levels were associated with higher fibrosis scores. Similar associations between viral activity, fibrosis progression, and hepatocellular injury have been widely documented [18,19].

A central finding of this study is the significant relationship between IL-6 elevations and markers of viral activity. IL-6 levels were not influenced by sex or age, confirming that IL-6 behaves as a demographic-independent inflammatory biomarker in chronic HBV infection. This observation is consistent with previous studies demonstrating that IL-6 levels are driven primarily by disease activity rather than host characteristics [20]. Although higher IL-6 levels were observed among HBeAg-positive individuals, this finding should be interpreted with caution because the HBeAg-positive subgroup was very small (*n* = 9), limiting statistical precision and generalizability. Nevertheless, IL-6 levels demonstrated a clear stepwise increase across low, moderate, and high HBV DNA groups, indicating their close association with HBV DNA levels.

Emerging evidence suggests that IL-6 is not only linked to HBV DNA but also strongly associated with viral antigens. Ghassan Haikel Abedallah Almosa et al. [21] demonstrated a significant association between IL-6 concentrations and both HBsAg and HBcAg levels, indicating that elevated IL-6 levels were associated with higher viral antigen levels in previous studies. This association may be relevant to hepatic inflammation and disease progression, although causality cannot be inferred from the present study. Under physiological conditions, IL-6 is expressed at low levels but is rapidly upregulated in response to infection-induced immune activation. Activated T lymphocytes and monocyte–macrophage lineages produce IL-6, which promotes T-cell proliferation and differentiation, thereby contributing to antiviral immune responses. However, previous experimental evidence has linked IL-6 signaling with hepatic inflammation and liver injury [22]. These experimental findings provide a biological context for the associations observed in our study. Several potentially relevant confounding factors, including antiviral treatment status, immune phase of chronic HBV infection, and coexisting inflammatory conditions, were not comprehensively evaluated in the present study. These factors may partly explain the modest variance explained by the regression model and should be addressed in future longitudinal investigations. The clinical relevance of IL-6 is further supported by studies linking elevated IL-6 to adverse outcomes in HBV infection. A significantly higher IL-6 elevation was observed in patients with moderate-to-severe chronic hepatitis B compared with those with mild disease, indicating that IL-6 reflects disease severity [23]. Similarly, a study demonstrated that elevated baseline IL-6 levels were connected with an increased risk of HBV-ACLF (HBV-related acute-on-chronic liver failure), suggesting that IL-6 may serve as an early predictive biomarker during severe acute exacerbations [24]. Although the predictive value of IL-6 requires validation in larger cohorts, routine IL-6 measurement at admission has been proposed to aid early risk stratification. Additional evidence indicates that IL-6, along with IL-10 and IL-17, plays a pivotal role in the immunopathogenesis of chronic HBV, with cytokine levels closely correlating with liver inflammation and viral load [25]. In the present study, PCA demonstrated distinct clustering of IL-6 and HBV DNA, separate from conventional biochemical markers of the liver, such as AST, ALT, and bilirubin. This pattern suggests that IL-6 levels were more closely associated with HBV DNA levels than with markers of hepatocellular injury or fibrosis. These findings were further supported by the correlation and regression analyses. Furthermore, Emhemed Abukhattala et al. [26] demonstrated in their study that IL-6 acted as a surrogate biomarker for monitoring HBV patients in clinical settings, reinforcing its utility in disease management. Beyond its role as a biomarker, IL-6 may represent a potential therapeutic target in chronic HBV infection because of its association with disease activity. However, regarding its involvement in both inflammatory and antiviral immune responses, further mechanistic and clinical studies are required before IL-6-targeted therapies can be considered in HBV management. The findings of this study should be interpreted in light of certain limitations. The study adopted a cross-sectional design, and important clinical variables, including antiviral treatment status, HBV disease phase, and comorbid inflammatory conditions, were not systematically collected. Future longitudinal studies with comprehensive clinical data are needed to validate and extend these findings.

## 5. Conclusions

In conclusion, serum IL-6 levels were significantly associated with HBV DNA levels and increased progressively with viral load. Multivariable analyses identified HBV DNA as the strongest independent predictor of IL-6, whereas conventional biochemical markers of liver injury and fibrosis showed weaker associations. These findings suggest that IL-6 may serve as an adjunct biomarker of disease activity in chronic hepatitis B. Nevertheless, more prospective longitudinal studies are required to validate its clinical utility and to clarify its role in HBV disease monitoring and management.

## Figures and Tables

**Table 1:** Clinical characteristics.

Characteristics	Total 293 (*n*_%)
Sex	
Male	160 (54.6)
Female	133 (45.3)
Age in years, median (IQR)	45 (23, 34–57)
Age group	
Below 20	4 (1.3)
21–30	36 (12.3)
31–40	68 (23.2)
41–50	73 (24.9)
51–60	55 (18.7)
Above 61	57 (19.4)
HBVDNA_(IU/mL)	
<2000	134 (45.7)
2001–200,000	138 (47.1)
>200,000	21 (7.2)
HBeAg (Positive/Negative)	9 (3)/284 (97)
T Bil_mg/dL	0.74 (0.68, 0.52–1.2)
D Bil_mg/dL	0.15 (0.2, 0.1–0.3)
AST_IU/L	27 (28, 20–48)
ALT_IU/L	24 (26, 16–42)
ALP_IU/L	72 (35, 59–94)
GGT_IU/L	23 (22, 15–37)
TP_g/dL	6.9 (1.10, 6.3–9.4)
Albumin_g/dL	3.8 (0.90, 3.3–4.2)
Globulin_g/dL	3, (0.6, 2.8–3.4)
B.urea_mg/dL	21 (10.50,16.5–27)
S. Creatinine_mg/dL	0.7 (0.3, 0.6–0.9)
Sodium_mmol/L	135 (4, 133–137)
Chloride_mmol/L	102 (6, 99–105)
PT	15 (2.02, 14.1–16.1)
INR	1.1 (0.28, 1–1.2)
APTT	28.4 (4.55, 26.5–31.1)
HBV DNA Log10_IU/mL	3.37 (1.29, 2.45–3.75)
IL6 Log10_pg/mL	2.38 (0.27, 2.21–2.49)
PLT_cu.mm	241,000 (103,500, 195,500–299,000)
FIB-4	1.09 (4.55, 0.67–1.69)
APRI	0.34 (0.46, 0.23–0.69)
PAGE-B	6 (6, 1–7)

Table Note: Values shown are *n (%)* unless otherwise indicated. Continuous variables are presented as median *(IQR)* because distributions were non-normal. IL-6 values were log10-transformed prior to analysis. HBV DNA categories reflect guideline-based clinical thresholds distinguishing inactive carriers (<2000 IU/mL), moderate replicative phase (2001–200,000 IU/mL), and high viral replication (>200,000 IU/mL). FIB-4 = fibrosis-4 index; APRI = AST-to-platelet ratio index; PAGE-B = Platelet, Age, Gender—hepatitis B predictive score for hepatocellular carcinoma in chronic hepatitis B patients. Platelet count represents absolute platelet numbers per mm^3^. HBeAg status reflects active viral replication.

**Table 2:** Comparison of Clinical and Biochemical Parameters Among HBV Viral Load Groups.

Characteristic	<2000 IU/mL *n* (%)	2000–200,000 IU/mL *n* (%)	>200,000 IU/mL *n* (%)	*p* Value
Sex				0.04
Male (*n* = 160)	70 (44.0)	73 (45.6)	17 (10.6)	
Female (*n* = 133)	64 (48.1)	65 (48.9)	4 (3.0)	
Total Bilirubin				0.001
Normal (*n* = 200)	95 (47.5)	99 (49.5)	6 (3.0)	
Abnormal (*n* = 93)	39 (41.9)	39 (41.9)	15 (16.1)	
Direct Bilirubin				0.001
Normal (*n* = 220)	112 (50.9)	101 (45.9)	7 (3.2)	
Abnormal (*n* = 73)	22 (30.1)	37 (50.7)	14 (19.2)	
AST				0.001
Normal (*n* = 173)	85 (49.1)	87 (50.3)	1 (0.6)	
Abnormal (*n* = 120)	49 (40.8)	51 (42.5)	20 (16.7)	
ALT				0.001
Normal (*n* = 195)	92 (47.2)	101 (51.8)	2 (1.0)	
Abnormal (*n* = 98)	42 (42.9)	37 (37.8)	19 (19.4)	
Blood Urea				0.04
Normal (*n* = 281)	130 (46.2)	133 (47.3)	18 (6.4)	
Abnormal (*n* = 12)	4 (33.3)	5 (41.7)	3 (25.0)	
Prothrombin Time				0.001
Normal (*n* = 210)	100 (47.6)	103 (49.0)	7 (3.3)	
Abnormal (*n* = 83)	34 (41.0)	35 (42.2)	14 (16.9)	
APTT				0.001
Normal (*n* = 242)	115 (47.5)	118 (48.8)	9 (3.7)	
Abnormal (*n* = 51)	19 (37.3)	20 (39.2)	12 (23.5)	
HBeAg Status				<0.001
Negative (*n* = 284)	133 (46.8)	138 (48.6)	13 (4.5)	
Positive (*n* = 9)	1 (11.1)	0 (0.0)	8 (88.9)	
FIB-4 Score				0.001
Low (*n* = 196)	100 (51.0)	89 (45.4)	7 (3.6)	
Intermediate (*n* = 68)	27 (39.7)	33 (48.5)	8 (11.8)	
High (*n* = 29)	7 (24.1)	16 (55.2)	6 (20.7)	
PAGE-B Score				0.01
Low (*n* = 274)	130 (47.4)	127 (46.4)	17 (6.2)	
Intermediate (*n* = 19)	4 (21.1)	11 (57.9)	4 (21.1)	
APRI Score				0.001
Low (*n* = 191)	94 (49.2)	96 (50.3)	1 (0.5)	
Intermediate (*n* = 77)	36 (46.8)	28 (36.4)	13 (16.9)	
High (*n* = 25)	4 (16.0)	14 (56.0)	7 (28.0)	

Table Note: Only variables showing statistically significant associations with HBV DNA categories are presented. Non-significant variables (age group, ALP, GGT, total protein, albumin, globulin, creatinine, sodium, potassium, chloride, platelet count, INR, and cirrhosis) are reported in Supplementary Table S2. HBV DNA categories were defined as <2000 IU/mL, 2000–200,000 IU/mL, and >200,000 IU/mL. *p* values were calculated using the chi-square test.

## Data Availability

The original contributions presented in this study are included in the article/Supplementary Material. Further inquiries can be directed to the corresponding author.

## Supplementary Materials

The following supporting information can be downloaded at: https://www.mdpi.com/article/10.3390/diseases14060209/s1, Table S1: Clinical Manifestation, Table S2: Clinical and biochemical variations across HBV viral load categories; Table S3: IL-6 Comparison Across Different Clinical Groups; Table S4: Multiple Linear Regression Analysis of Factors Associated with IL-6 Levels. Figure S1: Principal Component Analysis (PCA) of Viral and Biochemical Markers in Chronic Hepatitis B Patients.
