# Supplementary material for: Serum Interleukin-6 as an Inflammatory Biomarker Associated with HBV Viral Load in HBsAg-Positive Chronic Hepatitis B
Source: Diseases. 2026 Jun 10;14(6):209. doi: 10.3390/diseases14060209 (PMC13298012; doi:10.3390/diseases14060209)
Supplement: Supplementary file 1 [file diseases-14-00209-s001.zip › diseases-4291034-supplementary.pdf]

Supplementary Table S1. Clinical Manifestation

Parameter	n (%)
HBsAg positive	293(3.91)
Male	160(54.6)
Female	133(45.3)
HBeAg positive	9(3.07)
Male	8(2.7)
Female	1(0.34)
Clinical Manifestations	
Asymptomatic	108 (36.9)
Fatigue	128 (43.7)
Abdominal discomfort	76 (25.9)
Jaundice	11 (3.8)
Hepatomegaly	46 (15.7)
Loss of appetite	39 (13.3)
Nausea / GI discomfort	27 (9.2)

Note: Among the 7483 visitors, 293 patients were positive, and HBsAg seroprevalence was 3.91%, with 160 (54.6%) males and 133 (45.3%) females. HBeAg positivity was detected in 9 patients (3.07%), predominantly among males. Clinically, 36.9% of patients were asymptomatic, while fatigue (43.7%) was the most common symptom, followed by abdominal discomfort (25.9%). Other manifestations included hepatomegaly (15.7%), loss of appetite (13.3%), nausea or gastrointestinal discomfort (9.2%), and jaundice (3.8%), indicating that although many patients were asymptomatic, some presented symptoms related to hepatic involvement in chronic hepatitis B infection.xx

Supplementary Table S2. Clinical and biochemical variations across HBV viral load categories

**Characteristics**	**Total_n293**	**<2000IU/mL n(%)**	**2000–200,000IU/mL n(%)**	**>200,000IU/mL n(%)**	**P value**
Age group					
Below 20	4	2(50)	1(25)	1(25)	
21-30	36	22(61.1)	11(30.6)	3(8.3)	
31-40	68	30(44.1)	35(51.5)	3(4.4)	
41-50	73	38(52.1)	32(43.8)	3(4.1)	
51-60	55	23(41.8)	29(52.7)	3(5.5)	
above 61	57	19(33.3)	29(52.6)	8(14)	
Sex					
M	160	70(44)	73(45.6)	17(10.6)	
F	133	64 (48.1)	65(48.9)	4(3)	0.04*
Total bilirubin					
Normal	200	95(47.5)	99(49.5)	6(3)	
Abnormal	93	39(41.9)	39(41.9)	15(16.1)	0.001*
Direct bilirubin					
Normal	220	112(50.9)	101(45.9)	7(3.2)	
Abnormal	73	22(30.1)	37(50.7)	14(19.2)	0.001*
AST					
Normal	173	85(49.1)	87(50.3)	1(0.6)	
Abnormal	120	49(40.8)	51(42.5)	20(16.7)	0.001*
ALT					
Normal	195	92(47.2)	101(51.8)	2(1)	
Abnormal	98	42(42.9)	37(37.8)	19(19.4)	0.001*
ALP					
Normal	260	120(46.2)	123(47.3)	17(6.5)	
Abnormal	33	14(42.4)	15(45.5)	4(12.1)	0.5
GGT					
Normal	222	102(45.9)	106(47.7)	14(6.3)	
Abnormal	71	32(45.1)	32(45.1)	7(9.9)	0.5
T.protein					
Normal	287	133(46.3)	133(46.3)	21(7.3)	
Abnormal	6	1(16.7)	5(83.3)	0(0)	0.1
Albumin					
Normal	293	134(45.7)	138(47.1)	21(7.2)	
Abnormal					
Globulin					
Normal	152	69(45.4)	74(48.7)	9(5.9)	
Abnormal	141	65(46.1)	64(45.4)	12(8.5)	0.6
B.Urea					

Normal	281	130(46.2)	133(47.3)	18(6.4)	
Abnormal	12	4(33.3)	5(41.7)	3(25)	0.04*
S.Creatinine					
Normal	289	133(46)	136(47.1)	20(6.9)	
Abnormal	4	1(25)	2(50)	1(25)	0.3
Sodium	2		**2(100)**		
Normal	291	134(46)	136(46.7)	21(7.2)	
Abnormal	2		**2 (100)**		0.3
potassium					
Normal	282	129(45.7)	133(47.2)	20 (7.1)	
Abnormal	11	5(45.5)	5(45.5)	1(9.1)	0.9
Chloride					
Normal	231	103(44.6)	113(48.9)	15(6.6)	
Abnormal	62	31(50)	25(40.3)	6 (9.7)	0.4
PLT					
Normal	274	123(44.9)	110(40.1)	41(15)	
Abnormal	18	11(61.1)	3(16.7)	4(22.2)	
PT					
Normal	210	100(47.6)	103(49)	7(3.3)	
Abnormal	83	34(41)	35(42.2)	14(16.9)	0.001*
INR					
Normal	197	89(45.2)	98(49.7)	10(5.1)	
Abnormal	96	45(46.9)	40(41.7)	11(11.5)	0.1
APTT					
Normal	242	115(47.5)	118(48.8)	9(3.7)	
Abnormal	51	19(37.3)	20(39.2)	12(23.5)	0.001*
HBeAg					
Normal	284	133(46.8)	138(48.6)	13(4.5)	
Abnormal	9	1(11)	0(0)	8(89)	<0.001*
Cirrhosis					
Normal	264	125(47.3)	122(46.2)	17(6.4)	
Abnormal	29	9(31)	16(55.2)	4(13.8)	0.1
FIB score					
Low range	196	100(51)	89(45.4)	7(3.6)	
Intermediate range	68	27(39.7)	33(48.5)	8(11.8)	
High range	29	7(24.1)	16(55.2)	6(20.7)	0.001*
PAGE score					
Low range	274	130(47.4)	127(46.4)	17(6.2)	
Intermediate range	19	4(21.1)	11(57.9)	4(21.1)	
High range	0	0	0	0	0.01*
APRI score					
Low range	191	94(49.2)	96(50.3)	1(0.5)	0.001*

Intermediate range	77	36(46.8)	28(36.4)	13(16.9)	
High range	25	4(16)	14(56)	7(28)	

Table Note: HBV DNA categories were defined according to clinical relevance: <2000 IU/mL (inactive carrier range), 2000–200,000 IU/mL (intermediate viral replication), and >200,000 IU/mL (high replicative phase). *P* values denote comparisons among the three viral load groups (Chi-square). IL-6 values were log-transformed for analysis but are not included in this categorical comparison table.

Supplementary Table S3. IL-6 Comparison Across Different Clinical Groups

Comparison Group	n_1	n_2	Mean Rank (Group 1)	Mean Rank (Group 2)	U value	Z value	p-value
**Gender (Male vs Female)**	160	133	147.05	146.94	10631.5	-0.012	0.90
**Age (<45 vs >45 years)**	147	146	147.85	146.14	10606	-0.17	0.86
**HBeAg (Positive vs Negative)**	9	284	59.78	149.76	493	-3.137	0.002
**Cirrhosis (Yes vs No)**	29	264	152.00	146.45	3683	-0.335	0.70
**HBV DNA (Low vs High)**	134	21	71.56	119.12	543.5	-4.515	<0.001
**HBV DNA (Moderate vs High)**	138	21	75.25	111.24	793	-3.337	0.001
**HBV DNA (Low vs Moderate)**	134	138	110.17	162.07	5718	-5.439	<0.001

Note: Data are presented as Mann–Whitney U test results comparing serum IL-6 levels between groups, expressed as mean ranks for each group. A p-value < 0.05 was considered statistically significant.

Supplementary Table S4. Multiple Linear Regression Analysis of Factors Associated with IL-6 Levels

Variable	B (SE)	β	t	p-value
**Constant**	**2.036 (0.085)**	**—**	**23.924**	**<0.001**
**HBV DNA (log10 IU/mL)**	**0.076 (0.009)**	**0.458**	**8.337**	**<0.001**
**Age**	**0.000 (0.001)**	**-0.016**	**-0.300**	**0.765**
**TBil**	**-0.011 (0.013)**	**-0.124**	**-0.866**	**0.387**
**DBil**	**0.009 (0.026)**	**0.044**	**0.356**	**0.722**
**AST**	**~0.000**	**-0.196**	**-1.629**	**0.104**
**ALT**	**~0.000**	**0.194**	**2.031**	**0.043**
**ALP**	**~0.000**	**0.035**	**0.634**	**0.527**
**PT**	**0.000 (0.003)**	**0.007**	**0.098**	**0.922**
**INR**	**0.010 (0.013)**	**0.047**	**0.769**	**0.443**
**APTT**	**0.003 (0.003)**	**0.067**	**0.964**	**0.336**
**FIB-4**	**-0.003 (0.004)**	**-0.045**	**-0.710**	**0.478**

Note: The overall regression model was statistically significant ($F = 7.56$, $p < 0.001$), explaining 22.8% of the variance in IL-6 levels ($R^2 = 0.228$; adjusted $R^2 = 0.198$).

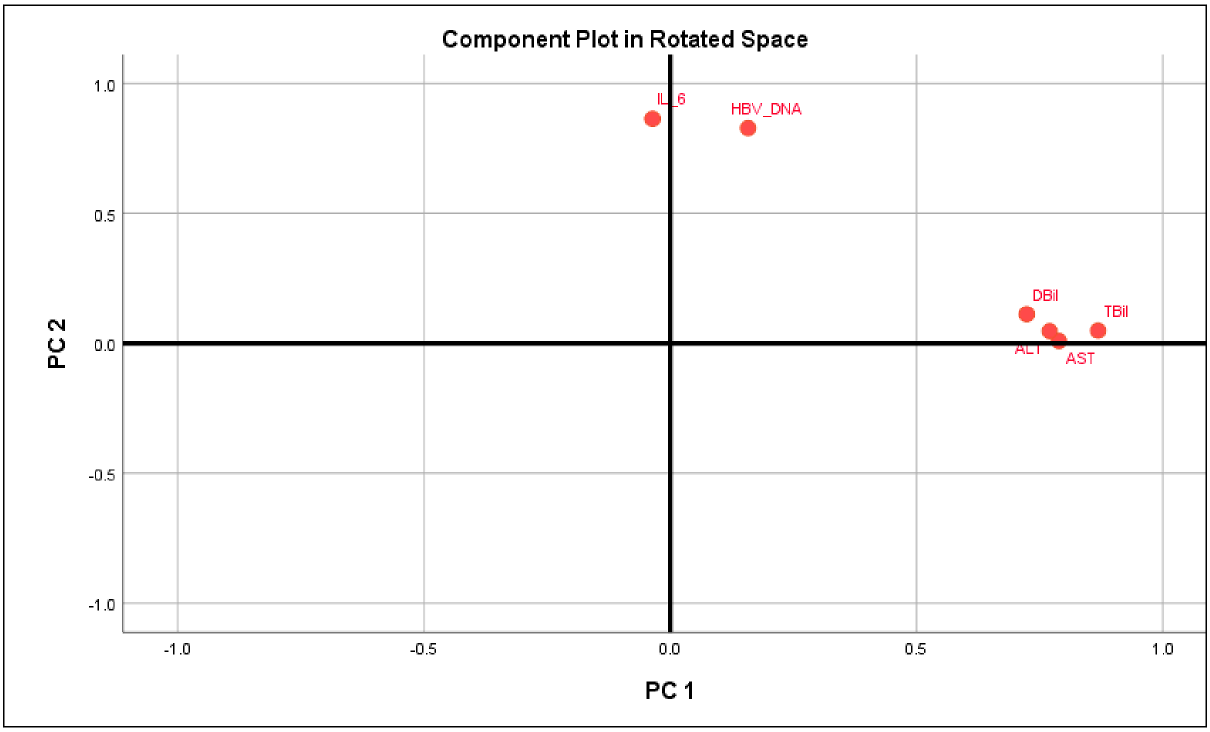

Supplementary Figure S1. Principal Component Analysis (PCA) of Viral and Biochemical Markers in Chronic Hepatitis B Patients.

Note: The PCA loading plot demonstrates clustering of HBV DNA and IL-6 along the viral-inflammatory component, while total and direct bilirubin cluster along a separate biochemical axis. This indicates that IL-6 is associated with viral replication rather than liver dysfunction.
